# A long-term ecosystem monitoring dataset from the ICP Integrated Monitoring network: biogeochemical data from 1977–2020 across 14 European countries

**DOI:** 10.1038/s41597-026-07181-8

**Published:** 2026-04-08

**Authors:** James Weldon, Wenche Aas, Barbara Albiniak, Algirdas Augustaitis, Ieva Baužienė, Camilla Capelli, Nicholas Clarke, Thomas Cummins, Heleen A. de Wit, Thomas Dirnböck, Ika Djukic, Karin Eklöf, Martin Forsius, Martyn Futter, Ulf Grandin, Sergei Gromov, Adéla Holubová Šmejkalová, Ricardo Ibañez, Iveta Indriksone, Sara Jutterström, Johannes Kobler, Heidi Koger, Angelika Kölbl, Andrzej Kostrzewski, Anna Koukhta, Pavel Krám, Robert Kruszyk, Esther Lasheras, Kairi Lõhmus, Mikołaj Majewski, Ulla Makkonen, Hampus Markensten, Rafael Miranda, Michael Mirtl, Filip Moldan, Giancarlo Papitto, Johannes Peterseil, Ainis Pivoras, Thomas Plha, Gisela Pröll, Pernilla Rönnback, Carolina Santamaría, Jesús Miguel Santamaría, Krzysztof Skotak, David Elustondo, Mercedes Valerio, Sarah Venier, Lieke E. Vlaar, Liisa Ukonmaanaho, Jussi Vuorenmaa, Nicole Wellbrock

**Affiliations:** 1 Swedish University of Agricultural Sciences (SLU), Uppsala, Sweden; 2 NILU, Norway, Oslo, Norway; 3 Chief Inspectorate for Environmental Protection, Warsaw, Poland; 4 Vytautas Magnus university, Kaunas, Lithuania; 5 State Scientific Research Institute Nature Research Centre, Vilnius, Lithuania; 6 University of Applied Sciences and Arts of Southern Switzerland (SUPSI), Mendrisio, Switzerland; 7 Norwegian Institute of Bioeconomy Research, Ås, Norway; 8 University College Dublin, Dublin, Ireland; 9 Norwegian Institute for Water Research NIVA, Oslo, Norway; 10 Environment Agency Austria, Vienna, Austria; 11 Finnish Environment Institute (Syke), Helsinki, Finland; 12 Institute of Geography RAS, Moscow, Russia; 13 Czech Hydrometeorological Institute, Prague, Czech Republic; 14 University of Navarra, Pamplona, Spain; 15 State Latvian Environment Geology and Meteorology Centre, Riga, Latvia; 16 Swedish Environmental Research Institute (IVL), Gothenburg, Sweden; 17 Ministry of Climate Estonia, Tallinn, Estonia; 18 Nationalparkverwaltung Bayerischer Wald, Grafenau, Germany; 19 Adam Mickiewicz University, Poznań, Poland; 20 Institute of Global Climate and Ecology Russia, Moscow, Russia; 21 Czech Geological Survey, Prague, Czech Republic; 22 Estonian Environmental Research Centre (EKUK), Tallinn, Estonia; 23 Finnish Meteorological Institute (FMI), Helsinki, Finland; 24 Comando Unità Tutela Forestale, Ambientale e Agroalimentare Carabinieri, Rome, Italy; 25 Federal Environment Agency Germany, Dessau-Roßlau, Germany; 26 Institute of Environmental Protection Poland, Warsaw, Poland; 27 National Institute for Public Health and the Environment (RIVM), Utrecht, Netherlands; 28 Natural Resources Institute Finland (Luke), Helsinki, Finland; 29 Thünen Institute, Braunschweig, Germany

## Abstract

The International Cooperative Programme on Integrated Monitoring of Air Pollution Effects on Ecosystems (ICP IM) presents a comprehensive long-term dataset of ongoing integrated ecosystem monitoring from European forested catchments. The dataset encompasses measurements from 46 monitoring stations across 14 European countries, with temporal coverage mostly extending from the early 1990s to 2020 (48 sites are currently active). The integrated monitoring approach applies over 20 monitoring subprogrammes to simultaneously measure physical, chemical, and biological properties across multiple ecosystem compartments including atmosphere, precipitation, throughfall, soil water, groundwater, runoff water, soil, vegetation, and biota. All measurements follow standardised protocols detailed in the ICP IM Manual, ensuring data quality and comparability across sites and time periods. The dataset supports research on ecosystem responses to air pollution, climate change impacts, and biogeochemical cycling. Data are available under a Creative Commons By Attribution (CC BY) licence, providing valuable long-term environmental monitoring data for the scientific community.

## Background & Summary

The ICP Integrated Monitoring programme was established under the UNECE Convention on Long-range Transboundary Air Pollution (CLRTAP) to monitor and assess the ecological effects of atmospheric pollutants on European ecosystems, with the principal challenge during its founding period in the early 1990s being so called “acid rain”, the transport and deposition of non-sea salt sulphur (S) that was at that time causing large scale forest die-off and lake acidification. As S emissions were successfully reduced in Europe, the focus of research using IM data in more recent years has shifted to investigating continued eutrophication caused by inorganic nitrogen (N) deposition, associated changes in biodiversity, recovery from acidification and declining heavy metal emissions. The programme addresses the critical need for long-term, standardised ecosystem monitoring data to understand cause-effect relationships between environmental pressures and ecosystem responses.

The IM concept involves the simultaneous measurement of physical, chemical, and biological parameters across different ecosystem compartments, at the same locations and over extended time periods. This approach enables quantification of mass balances, assessment of ecosystem recovery from pollution impacts, and the validation of dynamic ecosystem models. The monitoring sites are located in small, hydrologically well-defined catchments (maximum approx. 1000 ha) in relatively undisturbed areas such as nature reserves, ensuring minimal interference from local management activities (Table 1).

**Table 1:** Explanations of acronyms used.

Acronym	Full name
ICP IM	International Cooperative Programme on Integrated Monitoring of Air Pollution Effects on Ecosystems
UNECE	United Nations Economic Commission for Europe
CLRTAP	Convention on Long-range Transboundary Air Pollution
WGE	Working Group on Effects
NFP	National Focal Point
EMEP	European Monitoring and Evaluation Programme
ICP Waters	International Cooperative Programme on Assessment and Monitoring of the Effect of Air Pollution on Rivers and Lakes
ICP Forests	International Co-operative Programme on Assessment and Monitoring of Air Pollution Effects on Forests
GBIF	Global Biodiversity Information Facility
ISO	International Organisation for Standardisation
EUNIS	European Nature Information System
FAO	Food and Agriculture Organisation of the United Nations
WMO	World Meteorological Organisation

In practice, monitoring is organised into several compartmental subprogrammes^1^ that are interconnected either through the use of common parameters (cross-media flux approach) or by relying on the same or nearby monitoring stations (cause–effect approach). Measuring these fluxes and pools, as well as tracking the rate at which they change, is crucial for shaping effective, impact-based environmental policies.

**Fig. 1:**
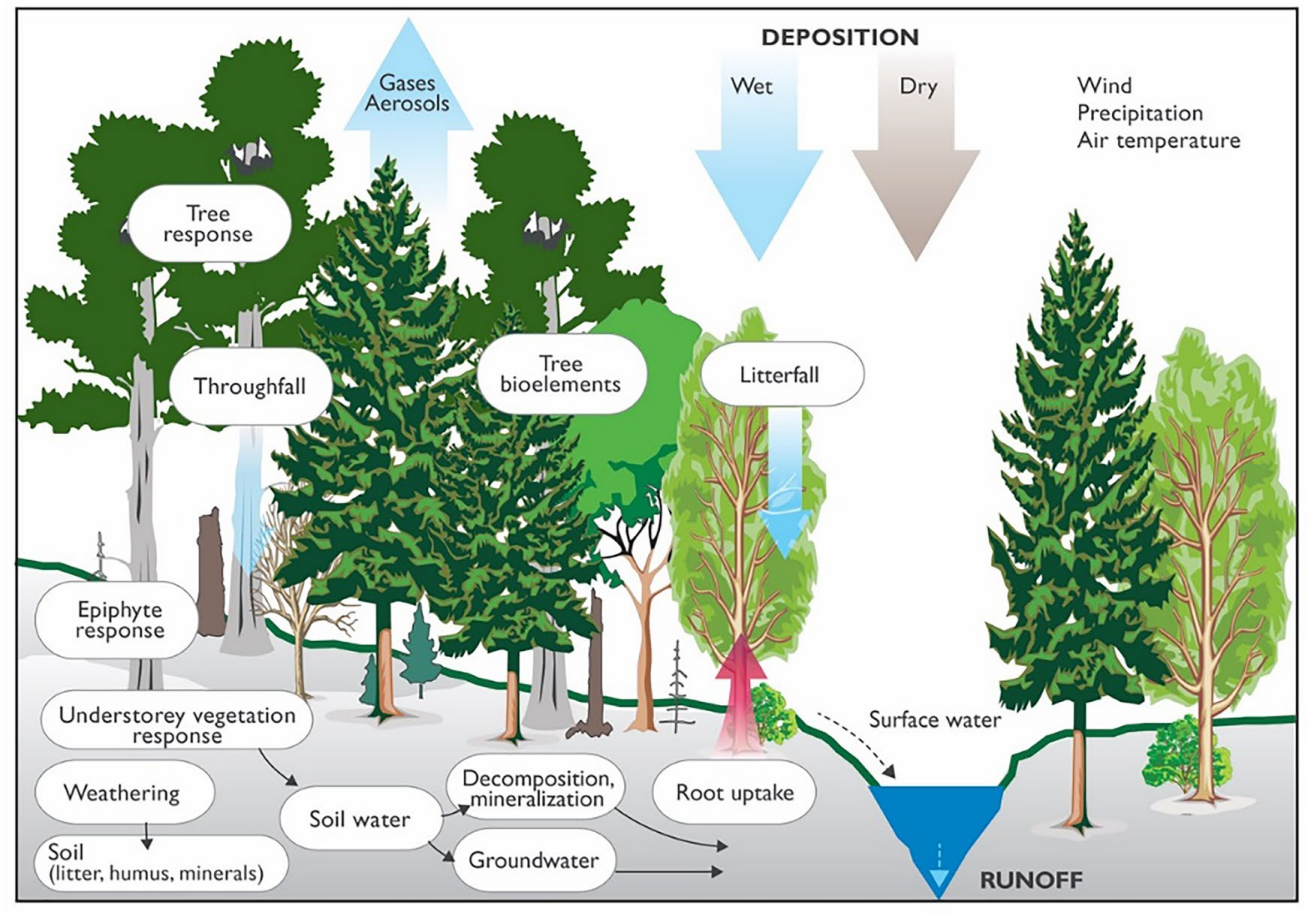
A conceptual scheme of a small catchment ecosystem showing main components (pools) and processes (fluxes) which are the objects of integrated monitoring (reproduced from the ICP IM Monitoring Manual).

A small catchment represents a sufficiently large unit to encompass all key interacting components: atmosphere and vegetation, plants and soils, bedrock and groundwater, streams or lakes, and the surrounding landscape (Fig. 1).

**Fig. 2:**
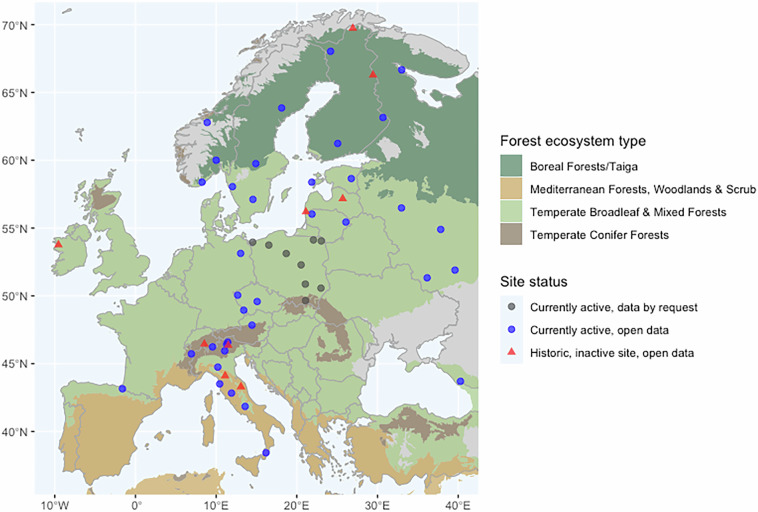
Map showing geographic distribution of ICP IM monitoring sites across Europe whose data are included in the published dataset. Blue dots indicate currently active sites providing open data, red triangles sites that are no longer active but where historical data are openly available. Grey dots indicate currently active sites where data are currently available by request only but metadata are open. Background coloured by forest ecosystem type^16^.

The ICP IM network currently comprises 48 active monitoring sites distributed across 15 European countries, representing major forest ecosystem types and pollution gradients across the continent^2^ (the open dataset covers 46 sites in 14 countries, includes historical data from several sites that are no longer operational, and note that the start dates of sites varies). Sites span Europe’s major forest types—from northern boreal forests to southern Mediterranean woodlands, and from eastern mixed forests to western temperate deciduous forests (Fig. 2)—capturing gradients in climate, geology, and pollution deposition. The programme is currently coordinated by the ICP IM Programme Centre at the Swedish University of Agricultural Sciences (SLU) and was previously hosted by the Finnish Environment Institute (SYKE).

The dataset described here represents one of the most comprehensive long-term integrated ecosystem monitoring efforts, with some sites providing continuous data records extending over 30 years. The data have been instrumental in assessing ecosystem recovery following reduced sulphur and heavy metal deposition, understanding nitrogen saturation processes, and evaluating ecosystem responses to climate change. The standardised methodology across all sites enables regional comparative analyses and supports the development of Europe-wide assessment models.

The reliability and value of ICP IM data have been demonstrated through a large body of scientific publications. The IM Annual Report^3^ includes a regularly updated summary of findings from over 60 key publications covering the following main areas of investigation (references here are examples, see the annual report for more):

- Input-output budgets: Site-specific budgets for S and total inorganic N, evaluating ecosystem retention and release patterns (total S is measured and the anthropogenic and sea-salt components calculated)^4^.
- Trend assessments: Long-term monitoring of deposition chemistry, runoff water quality, and climate variables documenting responses to emission reduction policies across the IM network^5^.
- Biological responses: biodiversity studies examining the effects of anthropogenic S and inorganic N deposition on vascular plant and bryophyte communities, and on epiphytic lichens^6–8^.
- Dynamic modelling and assessment of the effects of emission/deposition scenarios^9^.
- Heavy metals: Concentrations and fluxes of Cd, Pb, Hg, Cu, and Zn have been monitored to assess atmospheric deposition impacts and catchment retention^10^.
- Critical loads: Site-specific calculations linking modelled critical load exceedances of N and S with measured ecosystem impacts^11^.

## Methods

### Site selection and network design

ICP IM monitoring sites are selected according to criteria which aim to ensure representativeness and data quality. Sites must be located in hydrologically well-defined small catchments allowing for input-output budget calculations. The main selection criteria are as follows:

- Catchment areas, up to max.1000 hectares
- Hydrologically well-defined boundaries with quantifiable water fluxes
- Geologically homogeneous conditions where possible
- Minimal ongoing management activities (i.e. near-natural conditions)
- Representative of regional ecosystem conditions
- Minimum 50 km distance from any significant point pollution sources
- Suitable for long-term monitoring infrastructure

The type of permanent stations used for collecting monitoring data for different subprogrammes of the IM programme varies considerably (plots, groups of trees, sampling sites etc.), and the location of permanent stations for collecting monitoring data in the subprogrammes depends on the heterogeneity of soil, forest stands and vegetation. At least two stations for each subprogramme should be used at each IM site so that the variation of parameters within the monitoring site can be assessed.

Monitoring stations are typically placed in the most representative habitat of an IM site, such as a particular vegetation type or soil type. The stations for different subprogrammes are located close to one another to allow for wider ecosystem monitoring of a particular habitat. A group of these plots is called an intensive area and the station codes for each subprogramme belonging to the group is the same.

### Mandatory monitoring subprogrammes

Due to the very large number of variables covered by the different subprogrammes it would be impractical to give a full description of all the methods used here. Instead, we here give an overview of the scope and purpose of each subprogramme and refer the reader to the IM Monitoring Manual^1^ for a comprehensive description of the methodology. The manual is provided at the repository alongside the data.

#### Air Chemistry (AC)

Measurement of gaseous and particulate pollutants (SO₂, NO₂, O₃, particulate S, sum of NO_3_ in aerosols and gaseous nitric acid (HNO₃), and sum of gaseous NH_3_ and NH_4_ in aerosols, plus optional CO₂). These measurements provide information needed for assessing the input of air pollutants to the ecosystem due to long-range atmospheric transport, and air pollution concentrations can be compared to critical levels to assess the risk of direct effects on flora. Collectors are placed in freely exposed locations unsheltered by vegetation, although where dense forest makes this impossible the AC station can be located at the closest suitable location outside the IM site boundaries proper. The methods used are shared with EMEP^12^. Recommended measurement periods are daily to weekly for all components except ozone, which is monitored continuously with 1-hour average values. The reported temporal frequency in the database is a weekly to monthly aggregate depending on method.

#### Meteorology (AM)

Continuous measurement of air and soil temperature, precipitation, relative humidity, wind speed and direction, solar radiation, and derived parameters, with optional photosynthetically active radiation and UV-B radiation. The objectives are to describe climatic conditions at sites and their changes, to detect periods of extreme weather that stress tree vitality (freezing, late frost, drought, storm), and to provide input data for models predicting ecosystem responses. Data from neighbouring meteorological stations may be used if demonstrated to be representative for the IM site, though at minimum soil and ground temperatures must be measured at the site itself. Data are recorded at high temporal resolution (typically 1–30 minutes depending on parameter) and aggregated to daily, monthly, and annual values.

#### Epiphytes (EP)

Survey of epiphytic lichens on tree trunks (between 50 and 200 cm above ground) as biological indicators of, primarily, changes in acidifying deposition. Lichens are directly exposed to atmospheric gases and dissolved pollutants and possess different sensitivities to these factors; a sensitivity index for the whole lichen community can be calculated based on all species present. The ICP Forests grid method is recommended (a 10 × 50 cm grid, subdivided into five 10 × 10 cm quadrats, is attached to the trunk of sample trees and occurrence of each species within each quadrat is recorded) but equivalent methods may be applied. Surveys are performed on groups of 5–10 trees on each of 5–10 plots, using both permanent and temporary trees. Repeated every 1–5 years, preferably under dry conditions.

#### Foliage Chemistry (FC)

Annual collection and analysis of current and (for evergreen species) previous year foliage for nutrient elements and trace metals, providing information on the nutritional status of trees and, under some circumstances, the relative loading of pollutants. Elements that are measured: Ca, K, Mg, Na, N, P, S, Cu, Fe, Mn, Zn and TOC, with further elements as optional: Al, As, B, Cd, Cl, Cr, F, Mo, Ni and Pb. At least 8 trees of each main species (>10% of stems in plot) are sampled, with foliage taken from the upper third of the crown. Foliage sampling must be undertaken at the same phenological stage each year (late summer for deciduous species; dormant season for evergreens), and every five years sample trees are analysed individually to determine within-plot variability.

#### Litterfall Chemistry (LF)

Collection of falling leaf/needle litter using litter sacks (0.25–0.5 m² collecting area), with chemical analysis for nutrient content and mass quantification. Analyses of dead material (shed foliage) complement foliage chemistry (FC) and are important for assessing nutrient fluxes and the nutritional status of forest trees; comparisons between litter and foliage concentrations provide information about translocation and nutrient status. Elements that are measured include: Ca, K, Mg, Na, N, P, S, Cu, Fe, Mn, Zn and TOC, with further elements as optional: Al, As, B, Cd, Cl, Cr, F, Mo, Ni and Pb. Sampling is undertaken at least monthly, but samples can be pooled to periodic samples, and collectors are placed in connection with throughfall collectors to assess litterfall representatively for the catchment.

#### Precipitation Chemistry (PC)

Collection and chemical analysis of precipitation samples for major ions (SO₄, NO₃, NH₄, Cl, Ca, Mg, K, Na), pH, alkalinity, conductivity and precipitation amount, with the purpose of quantifying the input of wet deposition to the monitoring area. Further elements are optional: Al, As, Cd, Cr, Cu, Fe, Mn, Mo, Ni, Pb, Zn, total P, total S, total N. Collection uses bulk or wet-only precipitation samplers, and by simultaneous use of information from meteorology, air chemistry, throughfall and stemflow subprogrammes, total deposition to the site may be inferred. Precipitation samples are taken so that correct monthly values can be derived, and individual sampling periods should be as short as possible (weekly is recommended, and preferably daily).

#### Runoff Water Chemistry (RW)

Continuous or frequent (at least daily) measurement of stream discharge with regular (at least monthly) chemical analysis of runoff water, preferably at permanent weirs. Runoff is the main output of solutes from a catchment area, and element loss can be calculated by combining discharge measurements with concentration data. For establishing catchment budgets, flow-weighted sampling is recommended. Mandatory parameters are mainly related to acidification and are harmonised with the ICP Waters programme: alkalinity, SO₄, NO₃, Cl, dissolved organic C (DOC), pH, Ca, Mg, Na, K, labile Al, total N, NH₄, stream runoff, specific conductivity. Optional parameters are as follows: water temperature, total P, soluble reactive PO₄, total S, SiO₂, Fe, Mn, Cd, Zn, Cu, Ni, Pb, As, Cr, Mo, total Al, F, colour.

#### Soil Chemistry (SC)

Analysis of soil chemical properties including pH, total S, total P, total N, Ca, Mg, K, Na, Al, total organic C, and acidity, from different soil depths, reflecting primarily the acidification impacts of S and N deposition and the eutrophication impact of N. Also included are the derived variables CEC, base saturation and weathering, as well as descriptive variables, dry bulk density, stone content and particle size analysis. Mineral soil is sampled by fixed depth layers (0–5, 5–10, 10–20, 20–40, 40–80 cm) rather than pedogenic horizons to ensure comparability in time and space, with the humus layer sampled separately. Sampling typically every 5 years, with a one-off detailed vertical profile description during site establishment.

#### Soil Water Chemistry (SW)

Collection and analysis of soil solution at multiple depths (typically within and below the main rooting zone) using lysimeters or suction cup samplers. Soil water is intimately coupled with the chemical and biological processes in the upper soil layers and is sensitive to both acidification and nitrogen pollution, providing information on nutrient and toxicological conditions for plant roots and microbes. Measurements taken at least monthly, with at least 3 samplers per depth installed. Chemical parameters include: pH, conductivity, alkalinity, total N, NH₄, NO₃, total P, Ca, Mg, K, Na, total and labile Al, S, Cl and DOC. Optional parameters include: Mn, Fe, SiO₂, PO₄, total S, soil water flow and the derived variables cation/anion balance and total organic N.

#### Throughfall (TF)

Collection and analysis of precipitation that has passed through the forest canopy, enabling the total deposition input to the soil under the canopy to be determined. By comparing throughfall with open-area precipitation chemistry, canopy interception and the interaction and internal cycling of nutrients can be assessed. Samples are taken at least monthly, using at least 10 collectors to cover the large local variations in throughfall deposition. The collectors are recommended to be systematically placed around the vegetation or soil intensive monitoring plots. The collection surfaces are at a height of approximately 1 m to prevent contamination from the soil, with sample containers in a cool and dark place such as a pithole. Parameters include: precipitation amount, pH, specific conductivity, S, NO₃, NH₄, total N, Cl, Na, K, Ca, Mg, alkalinity and DOC. Optional parameters are: Al, Mn, Fe, As, Cd, Cr, Cu, Mo, Ni, Pb, Zn, total P and total S.

#### Vegetation (VG)

Detailed inventory of species composition on permanent intensive plots (40 m x 40 m although some flexibility is allowed), providing sensitive bioindication of changes in pollutant deposition or other atmospheric factors (e.g. warming) and data on the dynamics of tree biomass and canopy structure. Measurements include percentage cover by species in the tree/canopy layer, the shrub layer, the herb layer and the ground/moss layer. The understorey vegetation includes soil-growing vascular plants, bryophytes, and lichens, but not fungi or algae. Also measured is tree diameter and height measurements, and number of living, dead, and fallen trees by size class (allowing above ground biomass estimation). Sampling is undertaken between every 1–5 years depending on the stability/vulnerability of the vegetation (annual is recommended, especially when initiating monitoring so a time series can be established quickly); tree and shrub layers are observed every five years.

Each intensive vegetation plot has a number of subplots (typically 20–40 sample plots of 50 cm × 50 cm, distributed by stratified random sampling) where sampling is performed (i.e. per species percentage cover at each subplot is reported). Prior to a 2010 revision however, these subplot data were reported as a per species mean value over each quarter of the intensive plot (identified by the variable “TREE_OR_QUARTER”). Since the revision, this variable is used to identify the subplot (i.e. raw data of per species coverage at each subplot are reported, and “TREE_OR_QUARTER” is the subplot ID). Users must be careful as this is potentially confusing, but it was considered the least bad option for applying the revision as part of a timeseries.

### Optional monitoring subprogrammes

The programme includes numerous optional subprogrammes that may be implemented based on site-specific objectives and resources. Some of the subprogrammes are specifically addressed in other ICPs, for instance lake water chemistry and hydrobiology of lakes are regularly assessed by ICP Waters^13^. However, some of these are sparsely represented in the database (see Supplementary Table 1 for an overview of where and when these have been applied):

#### Aerial green algae (AL)

The aim is to obtain bioindication of changes in deposition of eutrophying substances, mainly N, by observing green algae on spruce needles, mainly *Pleurococcus vulgaris* (syn. *Protococcus viridis*), as the more N deposition, the thicker the algal cover and the more rapid their colonisation. This subprogramme can only be applied at sites where Norway spruce (*Picea abies*) is present, and observations are made annually in July-September on 15–20 small spruces. Using a magnifying lens the thickness of algal coverage is recorded on branches at approx. 160 cm height.

#### Inventory of birds (BB)

A census of birds breeding within the catchment area, using the territory mapping method on a grid of 50 m x 50 m meshes. The observation area is visited thoroughly 10 times during the breeding season, April-June, the data are analysed by species, and the number of pairs are calculated by the occurrence of territorial clusters and nests. The inventory is repeated every 3–5 years.

#### Tree bioelements and tree indication (BI)

Estimate the state and change in the amounts of chemical elements that are contained in the tree biomass, including the dead wood in a catchment - sometimes a more substantial pool than the soil pool. As well as elements, this programme monitors physical aspects of tree populations as biological indicators of pollution and other atmospheric change (recording species, diameter, height, vitality, crown diameter), and to keep a record of the trees as they relate to other subprogrammes, especially Throughfall (TF), Foliage chemistry (FC) and Litterfall (LF). Data are collected on sampling plots (preferably circular, 10 m radius) distributed across the catchment, repeated every 5 years. First, measurements of living and of standing and fallen dead wood are made, then biomass calculated by species specific functions using these measurement data. Finally, biomass per compartment is multiplied by locally valid element concentration parameters and scaled up to catchment level yield an estimate of the total amount of each element in the whole catchment, including an estimate of the standard error.

#### Forest damage, visual assessment of tree health indicators (FD)

Assessing tree defoliation and discolouration annually to obtain early quantitative indications of changes in the most important photosynthetically active parts of the trees. Assessments are made on over 100 trees per site, considering only the assessable crown (exposed to light), with trees selected from predominant, dominant or codominant classes. Defoliation is visually estimated and recorded in 5% steps compared to an imaginary, fully needled/leafed tree of the same type. Discolouration is similarly recorded as proportion of needles/leaves affected in 5% classes. Any apparent cause of defoliation such as evidence of fire or insect damage is also recorded.

#### Groundwater chemistry (GW)

Chemical composition of shallow groundwater, measuring the same variables as soil water chemistry (SW). Groundwater is monitored in observation tubes or springs, with sampling concentrated in the discharge area of the catchment and conducted at least 6 times per year, more frequently during snowmelt. Simple, non-electrical sampling equipment is used for soaking up groundwater through an observation tube, with a vacuum created by a hand pump used to suck groundwater into a sampler bottle.

#### Hydrobiology of lakes (LB)

Biological communities in lakes, providing indication of water quality changes. Chlorophyll α and phytoplankton primary production are sampled twice-monthly from spring to autumn, as well as macrozoobenthos samples (taken at least four times per year, with the first samples taken in spring shortly after ice breakup in areas with lake freeze-over, and not later than the end of May. The last samples of the year are taken in September-November).

#### Lake water chemistry (LC)

For sites with lake ecosystems, the same mandatory parameters as in runoff water chemistry (RW) are sampled, providing an integrated picture of fluxes from atmospheric and terrestrial environments. Samples are taken at the deepest point of the lake at multiple depths, 2–6 times per year.

#### Microbial decomposition (MB)

Changes in microbial activity can lower decomposition rates, leading to reduced nutrient uptake by the vegetation and increased amount of organic material. Measurements are based on field incubation of litter bags (containing 1 g of standard needles or a sheet of bleached alfa cellulose of a standard size) placed on the soil surface, covered with natural litter and retrieved for analysis after 1, 2 and 3 years. Bags are air-dried to halt decomposition and loss of weight calculated. Every fifth year, potential microbial activity (soil respiration, acid phosphatase activity, and net mineralization of nitrogen) is also determined under standard laboratory conditions on organic horizon samples.

#### Metal chemistry of mosses (MC)

Measuring the heavy metal content in moss tissues (*Pleurozium schreberi* or *Hylocomium splendens* preferred) as biomonitors of atmospheric deposition (As, Cd, Cr, Cu, Fe, Hg, Ni, Pb, Zn). Mosses are sampled in open areas at least 5 m from the nearest tree to avoid throughfall influence, with at least 3 composite samples collected every 5 years. One composite sample consists of five to ten subsamples spread around each sampling place, yielding about 2 liters of moss material for chemical analysis.

#### Hydrobiology of streams (RB)

Macrozoobenthos sampling as indicator of acidification or other stressors in streams, since invertebrate community composition responds to acid shocks and differs in species tolerance. Samples are collected twice a year (spring and autumn) using the kick-sampling method on hard bottoms with rapidly running water, with 3–6 replicate samples per site. Specimens are stored in ethanol and taxonomic identification performed to the best accuracy possible (species/genus level).

#### Stemflow (SF)

Chemical analysis of water running down tree stems (which can have an especially strong influence on epiphytic lichens present and on soil properties at the stem base), using the same analytic methods as the throughfall subprogramme (TF). Ten trees each of the most important species (i.e. >20% of the basal area in the plot) are monitored. The amount of stemflow varies markedly between species; trees with upward branch orientation (e.g. *Fagus sylvatica*) may contribute 10–40% of total stand precipitation, while those with drooping branches (such as Norway spruce, *Picea abies*) contribute <1%, so stemflow need not be measured for all species. Samples are taken at least monthly.

#### Vegetation structure and species cover (VS)

While the Vegetation (VG) subprogramme follows the development of a smaller defined area on a more frequent basis, this subprogramme establishes multiple circular 100 m² plots spread evenly across the whole catchment to follow any major changes in the structure, species composition and diversity of the plant communities of the site, also serving plant diversity monitoring needs. Percentage cover by species and vegetation layer (tree/canopy layer, the shrub layer, the herb layer and the ground/moss layer) is recorded, as in VG. Inventories are done only every 10–20 years (or after a major change such as a fire or large-scale bark beetle attack).

## Data Records

The dataset presented here^14^ is available at Researchdata.se, a data repository provided by the Swedish National Data Service: 10.5878/z376-2m63

The data are provided as comma separated value (CSV) files with UTF-8 encoding, with one file per subprogramme. The file for each subprogramme includes data from all sites and all years where this subprogramme has been performed.

Documentation files (CSV) are also provided with location data for monitoring sites, tables of codes used in the data, a list of parameters included in each subprogramme, and the ICP monitoring manual (in PDF format) for detailed descriptions of methods and background information.

The database is updated annually, and visitors to Researchdata.se will be advised that a more recent version is available and offered a link to the latest data, however the database as described in this paper will remain permanently available via the given DOI.

The column headers for the files depend on the subprogramme that is reported. There are three reporting formats or templates used. All chemical subprogrammes have a common reporting format (Table 2). Data from the biological subprogrammes: Vegetation VG, Aerial green algae AL and Forest damage FD are reported using the B1 reporting format (Table 3). Data from the rest of the biological subprogrammes: Trunk epiphytes EP, Tree bio elements and tree indication BI, Vegetation structure and species cover VS and Inventory of birds BB are reported using the B2 reporting format (Table 4). The column names and explanations of their meaning for each format is given in the three tables below (adapted from the IM Manual section 4.3.1).

**Table 2:** Data format for the chemical subprogrammes.

Column name	Description	Data type
**SUBPROG**	subprogramme code, file identifier	Text
**AREA**	country code and area number	Text
**INST**	2-letter code for institute	Text
**SCODE**	4-digit code for station	Numeric
**MEDIUM**	code for the sampled soil etc, indicated in each subprogramme (if biological see next rows)	Text
**MEDIUM_TAXONKEY_GBIF**	If the sample medium is biological (e.g. a tree) give GBIF Taxon ID	Numeric
**MEDIUM_NAME_GBIF**	If the sample medium is biological give GBIF scientific name	Text
**LISTMED**	medium code list (for soil codes and IM codes)	Text
**LEVEL**	measurement level	Numeric
**YYYYMM**	year month of the measurements	Date
**DAY**	day, normally not given	Text
**SPOOL**	spatial pool, number of devices/sampling points	Numeric
**SUBST**	substance code	Text
**LISTSUB**	list code for the parameter (DB or IM)	Text
**PRETRE**	pre-treatment code (for DB codes)	Text
**DETER**	determination code (for DB codes)	Text
**VALUE**	value in suggested unit, maximum 3 decimals	Numeric
**UNIT**	suggested units are given in each subprogramme, this is only verification	Text
**FLAGQUA**	data quality flag (see use of flags)	Text
**FLAGSTA**	status flag (2 letters reserved for the coding AM data) (see use of flags)	Text
**ADDIT**	only for subprogramme FC (see subprogramme FC)	Text

**Table 3:** biological data, format B1 (for subprogrammes VG, AL, FD).

Column name	Description	Data type
**SUBPROG**	subprogramme code, file identifier	Text
**AREA**	country code and area number	Text
**INST**	2-letter code for institute	Text
**SCODE**	4-digit code for station	Numeric
**MEDIUM_TAXONKEY_GBIF**	GBIF acceptedTaxonKey	Numeric
**MEDIUM_NAME_GBIF**	GBIF accepted scientificName	Text
**TREE_OR_QUARTER**	number of the sampled tree or (in subprogramme VG only) sample plot ID within intensive vegetation plot. Before 2010 reported as mean value for each quarter of the intensive vegetation plot.	Numeric
**YYYYMM**	year month of the measurements	Date
**SPOOL**	spatial pool, number of trees/sampling points	Numeric
**CLASS**	diameter/height classes (only subprogramme VG)	Numeric
**PARAM**	parameter code	Text
**PARLIST**	parameter list code	Text
**VALUE**	value in suggested unit, maximum 3 decimals	Numeric
**UNIT**	suggested units are given in each subprogramme, this is only verification	Text
**FLAGSTA**	status flag (2 letters reserved) (see use of flags)	Text
**DAMAGE**	only subprogramme FD, cause of damage	Text

**Table 4:** Biological data, format B2 (for subprogrammes EP, BI, VS and BB).

Column name	Description	Data type
**SUBPROG**	subprogramme code, file identifier	Text
**AREA**	country code and area number	Text
**INST**	2-letter code for institute	Text
**SCODE**	4-digit code for station	Numeric
**SIZE**	size of the observed area (only subprogramme BI and BB)	Numeric
**YYYYMM**	year month of the measurements	Date
**SPOOL**	spatial pool, number of trees/sampling points	Numeric
**MEDIUM_TAXONKEY_GBIF**	GBIF acceptedTaxonKey	Numeric
**MEDIUM_NAME_GBIF**	GBIF accepted scientificName	Text
**PFLAG**	permanent/non-permanent trees (only in subprogramme EP)	Text
**NAME_GBIF**	GBIF accepted scientificName	Text
**TAXONKEY_GBIF**	GBIF acceptedTaxonKey	Numeric
**TREE_ID**	Unique identifier for trees (only in BI)	Numeric
**CLASS**	diameter/height /decomposition/vitality classes (only in BI)	Numeric
**PARAM**	parameter code	Text
**PARLIST**	parameter list code	Text
**VALUE**	value in suggested unit, maximum 3 decimals	Numeric
**UNIT**	suggested units are given in each subprogramme, this is only verification	Text
**FLAGQUA**	quality flag (see use of flags below)	Text
**FLAGSTA**	status flag (2 letters reserved) (see use of flags below)	Text

### Use of flags

Two types of flags are used in the data reporting when necessary: data quality flag and status flag. The possible codes for flags are:

**Data quality flag** (FLAGQUA):

L = Less than detection limit (given as value) E = Estimated from measured value V = Species verified but no value given (in BB = Inventory of Birds)

For calculation of average values when values below detection limit are included (see Annex 7 of the IM Manual). Only if a primary value which is below detection limit is reported, the detection limit is given as the value and quality flag L is attached.

**Status flag** (FLAGSTA):

X = Arithmetic average, mean W = Weighted mean S = Sum M = Mode

Primary values are reported without a status flag. When averages and other calculated values are reported a status flag is attached. For calculation of average values, please see Annex 7 of the IM Manual.

### Data citation and attribution

When using ICP IM data in scientific publications, users should cite this data descriptor publication (following CC BY licence requirements for attribution). Users may wish to consider inviting collaboration with ICP IM scientists for complex applications. Although this is not required by the licence, it may be helpful to make use of the extensive background knowledge and wide-ranging expertise that exists within the programme. We would also encourage actively sharing any results and publications with the ICP IM community to support the future development of the programme. Please contact the ICP IM Programme Centre with any enquiries.

### Data interoperability

The ICP IM protocols have developed alongside the manuals of the sibling bodies under the UN Air Convention, and there is a considerable amount of overlap in methods and data formats with especially ICP Forests^15^ and ICP Waters^13^. Although some divergence occurs given the different aims of these bodies, and their partly independent development over time (and users should carefully check the relevant manuals) it is usually possible to combine data from multiple ICPs with fewer challenges than with unrelated datasets. The closest overlap with site design is with ICP Forests Level 2 sites, although these are not required to be catchment based and normally involve active forest management, in contrast to ICP IM sites.

### Data coverage and updates

The ICP IM database has previously been available through a “by request” model but has now transitioned to open data publication under a Creative Commons CC BY licence. As the data have previously been “by request”, it was necessary to obtain consent from data providers for the move to publication under a CC BY licence. A number of observations were requested to be omitted from the open publication, but the large majority of data submitted by currently active sites is included, and metadata are always open. There are also some cases where we have been unable to secure the necessary consent from countries which were active in the past (usually due to lack of continuity in personnel due to retirement, and subsequent uncertainty around who would have the appropriate authority to agree to open publication). This applies to sites in Belarus, Canada, Denmark and the United Kingdom and these data remain available by request. If you require any non-open data please contact the IM Programme Centre at im-database@slu.se specifying parameters, sites, and time periods of interest and these will be provided to you.

**Fig. 3:**
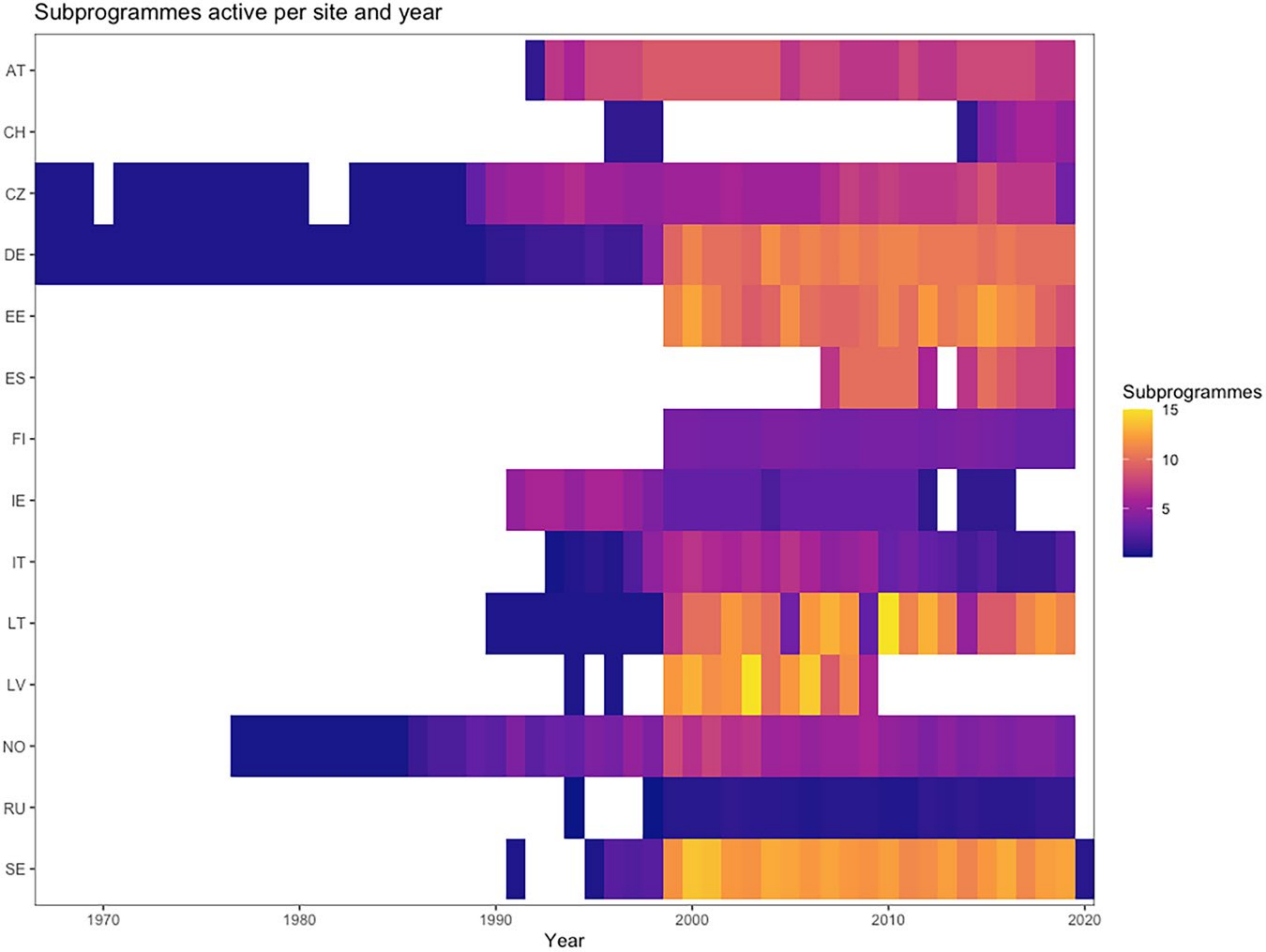
Heatmap overview of the mean number of subprogrammes per site across all countries and all years included in the open dataset.

An overview of the data coverage can be seen in Fig. 3. Two sites have meteorology data going back to 1967, long predating ICP IM. The earliest chemical data begin in 1977, but with the majority of sites starting to report data in the 1990s or later. For a complete overview of which site, year and subprogrammes are included see Supplementary Table 1.

## Technical Validation

### Quality assurance and quality control

Data quality is ensured through comprehensive QA/QC procedures as specified in the IM Manual, covering each stage from field sampling to laboratory analysis to database inclusion^1^. The general objective is that all data generated by the participating countries should be comparable on an objective basis, both consistent in time (to assess trends) and in space (for between-site and between-country comparisons). QA/QC procedures encompass all activities performed at the site and in the laboratory. Each National Focal Point is expected to ensure that good practice is followed and is required to submit a report on the QA/QC procedures followed by the laboratories involved, including detection limits for each analysed substance.

In the field, standard operational procedures are followed for all activities. All operators must be well-trained, and sites and equipment must be inspected at least once a year by the data originator. Field QA/QC routines include the use of field blanks and control samples, and specific requirements for sample transportation and storage. Materials that come into contact with samples must be chemically inert.

Laboratories involved in chemical analyses should be certified under a recognised accreditation system (e.g. EN 45001, ISO/IEC guide 25). In-laboratory quality control includes documentation of analytical performance, use of control charts, assessment of within-run precision and between-run accuracy, and ionic balance checks on individual samples. Between-laboratory quality control is maintained through participation in international ring tests (inter-comparison exercises). For water chemistry, target data quality objectives follow EMEP standards, including 10% accuracy or better for oxidised S and N, 15% accuracy or better for other components, and 90% data completeness of daily values. Suggested detection limits and target accuracies are specified for each determinant. Soil analyses include plausibility checks such as cation-anion balances, internal consistency checks (e.g. sand + clay + silt = 100%), and identification of outliers using standardised statistical criteria. For plant materials, accuracy is verified through analysis of certified reference standard samples and regular use of in-house standards.

### Data management and reporting

Data are collected by national monitoring organisations at three organisational levels: expert institutes collect and report primary data to National Focal Points (NFPs) and are responsible for data quality; the NFPs treat the data according to the IM Manual and report to the Programme Centre; and the Programme Centre collects, stores, and tests data quality. The reporting period is on a calendar year basis, with the previous year’s data typically due by December and results audited the following April. Data are submitted as Excel or comma-separated value (CSV) files following standardised reporting formats.

All data undergo systematic quality control before inclusion in the database, including verification (checking completeness, precision, and consistency of raw data, removing transcription errors) and validation (identification and assessment of outliers). The Programme Centre maintains the central database and produces annual reports on monitoring activities and data assessment.

## Supplementary information

Supplementary Information

## Data Availability

The dataset presented here^14^ is available at the Swedish National Data Service data repository Researchdata.se as CSV files (one per subprogramme) alongside documentation files with geographic information, codes used in data files and a PDF of the full ICP IM monitoring manual: 10.5878/z376-2m63.

## Code availability

Python code used to screen data submissions and flag errors can be found at Figshare: 10.6084/m9.figshare.31422386.

## References

[1] ICP IM Programme Centre. *ICP IM Manual for Integrated Monitoring: Convention on Long-Range Transboundary Air Pollution of the UNECE International Cooperative Programme on Integrated Monitoring of Air Pollution Effects on Ecosystems*. https://pub.epsilon.slu.se/38969/1/kuren-weldon-j-et-al-20251201.pdf (2025).

[2] Grennfelt, P. *et al*. Acid rain and air pollution: 50 years of progress in environmental science and policy. *Ambio* **49**, 849–864, 10.1007/s13280-019-01244-4 (2020). 31542884 10.1007/s13280-019-01244-4 PMC7028813

[3] *34th Annual Report 2025: Convention on Long-Range Transboundary Air Pollution: International Cooperative Programme on Integrated Monitoring of Air Pollution Effects on Ecosystems*, 10.54612/a.3bg8n3vnor (2025).

[4] Vuorenmaa, J. *et al*. Long-term sulphate and inorganic nitrogen mass balance budgets in European ICP Integrated Monitoring catchments (1990–2012). *Ecol. Indic.* **76**, 15–29, 10.1016/j.ecolind.2016.12.040 (2017).

[5] Vuorenmaa, J. *et al*. Long-term changes (1990-2015) in the atmospheric deposition and runoff water chemistry of sulphate, inorganic nitrogen and acidity for forested catchments in Europe in relation to changes in emissions and hydrometeorological conditions. *Sci. Total Environ.* **625**, 1129–1145, 10.1016/j.scitotenv.2017.12.245 (2018). 29996410 10.1016/j.scitotenv.2017.12.245

[6] Dirnböck, T. *et al*. Forest floor vegetation response to nitrogen deposition in Europe. *Glob. Chang. Biol.* **20**, 429–440, 10.1111/gcb.12440 (2014). 24132996 10.1111/gcb.12440

[7] Dirnböck, T. *et al*. Currently legislated decreases in nitrogen deposition will yield only limited plant species recovery in European forests. *Environ. Res. Lett.* **13**, 125010, 10.1088/1748-9326/aaf26b (2018).

[8] Weldon, J. & Grandin, U. Weak recovery of epiphytic lichen communities in Sweden over 20 years of rapid air pollution decline. *Lichenologist* **53**, 203–213, 10.1017/S0024282921000037 (2021).

[9] Holmberg, M. *et al*. Modelling study of soil C, N and pH response to air pollution and climate change using European LTER site observations. *Sci. Total Environ.* **640–641**, 387–399 (2018). 29860010 10.1016/j.scitotenv.2018.05.299

[10] Eklöf, K. *et al*. Trends in mercury, lead and cadmium concentrations in 27 European streams and rivers: 2000-2020. *Environ. Pollut*. 124761, 10.1016/j.envpol.2024.124761 (2024). 10.1016/j.envpol.2024.124761 39154885

[11] Forsius, M. *et al*. Assessing critical load exceedances and ecosystem impacts of anthropogenic nitrogen and sulphur deposition at unmanaged forested catchments in Europe. *Sci. Total Environ.* **753**, 141791, 10.1016/j.scitotenv.2020.141791 (2021). 32890870 10.1016/j.scitotenv.2020.141791

[12] EMEP manual for sampling and chemical analysis. https://emep-ccc.nilu.no/manual.

[13] Gundersen, C. B. *et al*. *ICP Waters Programme Manual*. https://www.icp-waters.no/publications/#icpwmanual (2025).

[14] Weldon, J. *et al*. The international cooperative programme on integrated monitoring of air pollution effects on ecosystems (ICP IM), 10.5878/z376-2m63 (2026).

[15] UNECE ICP Forests Programme Coordinating Centre. *Manual on Methods and Criteria for Harmonized Sampling, Assessment, Monitoring and Analysis of the Effects of Air Pollution on Forests*. https://www.icp-forests.net/monitoring-and-research/icp-forests-manual (2022).

[16] Olson, D. M. *et al*. Terrestrial ecoregions of the world: A new map of life on earth. *Bioscience* **51**, 933 (2001). 10.1641/0006-3568(2001)051[0933:TEOTWA]2.0.CO;2.

